# Retrospective Cohort Study Comparing Complications, Readmission, Transfusion, and Length of Stay of Patients Undergoing Simultaneous and Staged Bilateral Total Hip Arthroplasty

**DOI:** 10.1111/os.12617

**Published:** 2020-01-20

**Authors:** Sheng‐jie Guo, Hong‐yi Shao, Yong Huang, De‐jin Yang, Han‐long Zheng, Yi‐xin Zhou

**Affiliations:** ^1^ Department of Adult Reconstructive Surgery, Beijing Jishuitan Hospital Fourth Clinical College of Peking University, Jishuitan Orthopaedic College of Tsinghua University Beijing China

**Keywords:** Complication, Length of stay, Staged surgery, Simultaneous surgery, Total hip arthroplasty

## Abstract

**Objectives:**

To determine whether the rates of postoperative complications, rate of readmission, cumulative transfusion volume, and length of stay (LOS) differ between simultaneous total hip arthroplasty (THA) and staged bilateral THA and to assess whether the length of the interval between staged procedures influences surgery outcome.

**Methods:**

This was a retrospective cohort study comparing the rate of postoperative complications, readmission, cumulative transfusion volume, and LOS between simultaneous THA and staged bilateral THA in our hospital's registration database. The inclusion criteria is listed as follows: patients who underwent bilateral primary THA between January 2011 and January 2015 with minimum 3‐month follow‐up; simultaneous bilateral THA; staged bilateral THA; postoperative complications, readmission, cumulative transfusion volume, length of stay of the patients and the influence of the interval between stages of bilateral THA on the outcome above; and retrospective cohort study. Finally, a total of 1145 patients, including simultaneous bilateral THA in 863 patients (1726 hips) and staged bilateral THA in 282 patients (564 hips), were eligible for the present study. The patients were divided into three groups according to the interval time (≤30 days, 30–90 days, >90 days) between the two stages of bilateral THA and we compared postoperative complications, readmission rates, cumulative transfusion volume, and LOS for the three groups. All patients’ medical records and outpatient notes were reviewed to extract preoperative data, perioperative complications, readmission, cumulative transfusion, and LOS. Preoperative information included patients’ age, sex, diagnosis, body mass index, and American Society of Anesthesiologists (ASA) classification. Perioperative complications were sorted into two groups: (i) medical complications included cardiovascular, pulmonary, neurological, digestive, and urologic system complications, along with other miscellaneous issues; and (ii) surgical complications included dislocation, superficial wound infection, hematoma, deep periprosthetic joint infection, and nerve palsy. Patients who failed to come back to visit our hospital in the postoperative 3 months were followed up by telephone, at which point we inquired about any postoperative complications and readmission.

**Results:**

Simultaneous THA was performed more often in younger men, and patients in the simultaneous group had fewer major medical complications (excluding venous thromboembolism), fewer surgical complications, and shorter hospital stays; however, patients in the simultaneous group were likelier to have a higher transfusion rate than patients in the staged group. Among patients in the staged group, there were no differences for differing time intervals, except that patients with a between‐stage interval of ≤30 days required more blood transfusions.

**Conclusion:**

With careful patient assessment and selection, simultaneous bilateral THA is a safe procedure, and has lower rates of surgical and major medical complications than staged bilateral THA.

## Introduction

Total hip arthroplasty (THA) is deemed one of the most successful procedures in the twentieth century due to the reported high levels of satisfaction and significant improvements postoperatively for patients in daily activities and function^1^. Many patients with avascular femoral head necrosis, developmental dysplasia, ankylosing spondylitis, or rheumatoid arthritis require bilateral THA because these diseases often affect both hips. Moreover, Siraj *et al*.^2^ report that patients undergoing unilateral index THA as a result of osteoarthritis may need contralateral THA within 10 years. In 1971, Charnley first demonstrated the feasibility of simultaneous (one‐stage) bilateral THA^3^. Since then, several studies have reported the positive outcome of simultaneous bilateral THA^4^, ^5^, ^6^. Stavrakis *et al*. reported that bilateral THA had similar complication rates to unilateral total hip arthroplasty and could be a safe procedure in appropriately selected patients^6^.

Bilateral THA has grown in popularity^7^, but the choice between simultaneous and staged bilateral THA remains controversial. Simultaneous bilateral THA may reduce the cost and length of hospital stay because anesthesia is needed only once^8^. Other researchers^9^ have shown simultaneous bilateral THA to be more effective than staged THA because it allows for substantial improvement in hip motion and better functional recovery. Aghayev *et al*.^10^ demonstrated that patients who underwent simultaneous bilateral THA have a lower rate of major systemic complications when compared with two‐staged bilateral THA in a study enrolling 1819 patients. Parvizi*et al*.^11^ demonstrated that there is no difference in systemic complications between unilateral THA and simultaneous bilateral THA. According to Rasouli *et al*.^7^, simultaneous bilateral THA are often performed on younger and male patients with fewer preoperative comorbidities, and patients with two‐staged bilateral THA theoretically experience twice the risk of a single surgery.

However, some researchers believe that simultaneous bilateral THA poses greater risks to patients, including a higher transfusion rate and a higher rate of postoperative complications^12^, ^13^. Ritter *et al*.^12^ found that the risks of myositis ossificans and phlebitis were higher, and the postoperative hip range of motion was lower in the patients who underwent simultaneous bilateral THA when compared with unilateral THA. Ritter *et al*.^12^ also reported that the length of hospital stay of patients who underwent unilateral THA was almost 1 week less than in patients who underwent simultaneous bilateral THA. In addition, the duration of the operation and blood loss in the simultaneous bilateral THA group were on average twice that in the unilateral THA group. Berend *et al*.^13^ demonstrated significantly higher rates of inpatients complications and more adverse events in patients with one‐staged bilateral THA in the lateral decubitus position. The authors also found greater transfusion volume, more patients failing to reach postoperative rehabilitation goals, increased need to transfer to rehabilitation facilities, and higher rates of subsequent hip surgery but 28% reduction in hospital reimbursement and 15% reduction in surgeon reimbursement.

Tsiridis *et al*.^14^ compared dislocation rates and thromboembolic complications with pooled data in a systematic review and meta‐analysis and found that no difference existed as to dislocation rates and thromboembolic complications between simultaneous and staged bilateral THA. In another meta‐analysis^15^ comparing simultaneous versus staged bilateral THA, the incidences of major postoperative complications such as cardiovascular or pulmonary problems and deep venous thrombosis (DVT) were lower in the simultaneous group than in the staged group. There were no differences between the two groups regarding other issues. For ethical reasons, it was difficult to carry out a randomized study of major postoperative complications, so only one randomized controlled study^16^ of a limited number of cases has been conducted. Other than some studies using data from registries with large numbers of cases, all other retrospective studies involved only a limited number of cases.

New technologies and perioperative protocols have been introduced and have evolved in last decade; they may influence the outcome of patients undergoing simultaneous and staged bilateral THA. Moreover to our knowledge, there have been no reports comparing simultaneous and staged bilateral THA performed at a single institution with a large volume of cases. Therefore, we conducted this study to answer the following questions:

1. Are the rates of postoperative complications different between simultaneous THA and staged bilateral THA groups?

2. Is the rate of postoperative readmission different between simultaneous THA and staged bilateral THA groups?

3. Is the postoperative cumulative transfusion volume different between simultaneous THA and staged bilateral THA groups?

4. Is the postoperative length of stay (LOS) different between simultaneous THA and staged bilateral THA groups?

5. Does the interval time between stages of bilateral THA influence postoperative complications, readmission, transfusion, and LOS of the patients?

Our hypotheses are that the rates of postoperative complications, readmission, cumulative transfusion volume, and LOS differ between simultaneous THA and staged bilateral THA and that the length of the interval time between staged procedures influences the rates of postoperative complications, readmission, cumulative transfusion volume, and LOS.

## Patients and Methods

### *Patient Data*

This was a retrospective cohort study comparing the rates of postoperative complications, readmission, transfusion, and LOS between simultaneous THA and staged bilateral THA in our hospital's registration database. The inclusion criteria was as follows: (i) patients who underwent bilateral primary THA between January 2011 and January 2015 with minimum 3‐month follow‐up; (ii) simultaneous bilateral THA; (iii) staged bilateral THA; (iv) postoperative complications, readmission, cumulative transfusion volume, LOS of the patients, and the influence of the interval between stages of bilateral THA on the outcome above; and (v) retrospective cohort study. Exclusion criteria: (i) patients with simultaneous total knee arthroplasty; (ii) patients with anemia or blood coagulation disorders preoperatively; and (iii) patients with bone tumors.

Finally, a total of 1145 patients, including simultaneous bilateral THA in 863 patients (1726 hips) and staged bilateral THA in 282 patients (564 hips), were eligible for the present study. Informed consent was obtained from all patients or their family members, and the research ethics committee of our hospital approved our study.

### *Surgery Process*

All the THA were performed with combined spinal epidural or general anesthesia according to the judgment of the anesthesiologist in lateral decubitus position. The incision was made through the posterolateral approach, and the tensor fasciae latae and gluteus maximus were split in the line of the muscle fiber. The external rotators and the joint capsular were incised, followed by resection of the labrum, ligamentum capitis femoris, and other fat or fibrous tissue and careful preparation of the acetabulum and femoral canal. The acetabular cup and femoral stem were implanted, ensuring optimal position and orientation.

### *Perioperative Management Protocol*

Although our protocol for perioperative management has evolved since our surgical volume began increasing in 2000, both consecutive cohorts in our study underwent surgery starting in 2011, and by that point, the protocol was the same for all patients. Patients were selected to undergo either simultaneous THA or staged bilateral THA on the basis of each patient's general status and after discussion between the medical team and the individual patient. Patients were admitted to hospital 1 day before surgery after medical clearance. A urinary catheter was placed, and one dose of antibiotics was infused preoperatively. For all patients, the posterolateral approach was used, cementless prostheses were used, and surgical drains were placed. Autologous blood salvage was carried out for every procedure.

After surgery, we used sterile draping to cover the wound. We removed the drain and the urinary catheter and changed the wound dressing on the day after surgery. Antibiotics were given for the 48 h after surgery to prevent infection; patients were also given low‐molecular‐weight heparin to prevent DVT and pulmonary embolism (PE). The rehabilitation protocol was the same in both groups, and patients were encouraged to engage in partial weight‐bearing on the day after surgery. Our hospital's medical internists managed postoperative medical issues. All patients were instructed to come back for follow‐up evaluation 3 months after surgery.

### *Data Collection and Follow‐up*

We reviewed all patients’ medical records and outpatient notes to extract data on preoperative status, perioperative complications, readmission, cumulative transfusion, and LOS. Preoperative information included patients’ age, sex, diagnosis, body mass index, and American Society of Anesthesiologists (ASA) classification. Patients who failed to come back to visit our hospital 3 months postoperatively were followed up by telephone and we inquired about any postoperative complications and readmissions.

### *Complications*

We divided perioperative complications into two groups: (i) medical complications, including cardiovascular, pulmonary, neurological, digestive, and urologic system complications, along with other miscellaneous issues; and (ii) surgical complications, including dislocation, superficial wound infection, hematoma, deep periprosthetic joint infection, and nerve palsy. Medical complications that occurred in the same patient during either procedure in the staged group were added and compared. Surgical complications were recorded for each hip for both the simultaneous and staged groups. If the patients had leg swelling or pain, we used deep vein ultrasonography to exclude DVT. DVT was diagnosed on the basis of ultrasound findings and was treated with an anticoagulant. PE was confirmed with spiral CT in patients with suspicious symptoms. More complications indicates poor outcomes and safety of the procedure.

We also categorized medical complications into two groups: (i) major medical complications, including myocardial infarction, frequent angina, cardiac arrhythmia, pulmonary infection, exacerbation of chronic obstructive pulmonary disease, stroke, stress ulcers and peptic ulcers, and bleeding, all of which increase the risk of death and require a major change in medical management; and (ii) minor medical complications, including urinary retention or infection, transient hypoxia, and gastrointestinal dysfunction.

### *Readmission, Cumulative Transfusion, and Length of Stay*

Patients who returned to the hospital for any reason within 90 days of original discharge were counted as having been readmitted. Cumulative transfusion and LOS were also added for both procedures in the staged group. High readmission rates and prolonged LOS indicate poor safety and greater cost of the procedure. More cumulative transfusion implies more blood loss from the procedure.

We also divided patients into three groups according to the time between the two stages of bilateral THA. The first group had an interval of <30 days between stages, the second group had an interval of 30 to 90 days, and the third group had an interval of >90 days. We compared postoperative complications, readmission rates, transfusion rates, and LOS for the three groups.

### *Statistical Analysis*

We used SPSS (version 17.0; IBM, Armonk, NY, USA) to carry out the statistical analyses. The Kolmogorov–Smirnov test was performed to examine the normality of the continuous data. The two‐sided nonparametric Wilcoxon rank‐sum (Mann–Whitney *U*) test was performed because the continuous data were not normally distributed. Ordinal categorical variables (ASA classification) were analyzed using nonparametric Mann–Whitney *U*‐tests. The χ^2^ or the Fisher exact test were used for all other categorical data. Cumulative transfusion and LOS between the three subgroups within the staged bilateral THA group were compared using a one‐way analysis of variance. Post hoc comparison was performed using the Scheffé test. The significance level was set at *P* < 0.05.

## Results

### *Patients’ Demographic Data*

The diagnosis profile differed somewhat between the two groups (*P* = 0.006, Table 1). The staged group had more cases of osteoarthritis, but the simultaneous group had more cases of avascular necrosis of the femoral head. Although the difference in preoperative ASA classification was not statistically significant (*P* = 0.064) between the two groups, the staged group had more patients with a higher preoperative class, which reflected how medical status influenced the decision between simultaneous and staged bilateral THA.

**Table 1:** Patient demographics

Parameters	Simultaneous group	Staged group	*P‐*value
Number of patients (hips)	863 (1726)	282 (564)	
Age (years)*	49 (41–56)	52.5 (44.75–60)	<0.001
Sex†			<0.001
Male	604 (70.0)	152 (53.9)	
Female	259 (30.0)	130 (46.1)	
BMI (kg/m^2^)*	24.69 (22.68–27.17)	24.79 (22.34–27.34)	0.646
ASA classification†			0.064
1	265 (30.7)	76 (27.0)	
2	589 (68.2)	194 (68.8)	
3	9 (1.0)	12 (4.3)	
Diagnosis†			0.006
AVN	580 (67.2)	158 (56.0)	
OA	219 (25.4)	101 (35.8)	
AS	53 (6.1)	19 (6.7)	
RA	11 (1.3)	4 (1.4)	

* Continuous variables which are not normally distributed and presented as medians with interquartile ranges in parentheses.

† Categorical variables which are presented as frequency with percent in parentheses.

AS, ankylosing spondylitis; ASA, American Society of Anesthesiologists; AVN, avascular necrosis; BMI, body mass index; OA, osteoarthritis; RA, rheumatoid arthritis

The patients in the simultaneous group were much younger (*P* < 0.001) and the group had more males (*P* < 0.001). However, body mass index values were similar between the two groups (*P* = 0.646, Table 1).

### *Medical Complications*

There were no statistically significant differences between the simultaneous THA and staged THA groups for medical complications of all systems, PE, and DVT (Table 2).

**Table 2:** Perioperative and postoperative complications

Parameters	Simultaneous group	Staged group	*P‐*value
Number of patients (hips)	863(1726)	282 (564)	
Number of medical complications*			
Cardiovascular	15 (1.7)	10 (3.5)	0.071
Pulmonary	17 (2.0)	8 (2.8)	0.387
Neurological	6 (0.7)	4 (1.4)	0.445
Digestive	23 (2.7)	10 (3.5)	0.443
Urological	13 (1.5)	2 (0.7)	0.471
PE	2 (0.2)	2 (0.7)	0.255
DVT	42 (4.9)	15 (5.3)	0.762
Miscellaneous	13 (1.5)	6 (2.1)	0.660
Number of major medical complications without VTE*	41 (4.8)	23 (8.2)	0.031
Number of major medical complications including VTE*	78 (9.0)	33 (11.7)	0.189
Number of minor medical complications*	44 (5.1)	16 (5.7)	0.707
Number of surgical complication*			
Superficial infection	11 (0.6)	9 (1.6)	0.034
Deep infection	3 (0.2)	1 (0.2)	1.000
Hematoma	0 (0)	1 (0.2)	0.246
Nerve palsy	1 (0.1)	0 (0)	1.000
Dislocation	1 (0.1)	3 (0.5)	0.049
Number of total surgical complications*	16 (0.9)	14 (2.5)	0.005
Number of readmissions*	14 (1.6)	5 (1.8)	1.000
Cumulative number of transfusions (units)†	4 (4–6)	4 (0–6.5)	<0.001
LOS (days)†	11 (8–14)	20 (16–24)	<0.001

* Categorical variables which are presented as frequency with percent in parentheses.

† Continuous variables which are not normally distributed and presented as medians with interquartile ranges in parentheses.

DVT, deep venous thrombosis; LOS, length of stay; PE, pulmonary embolism; VTE; venous thromboembolism

Fifteen patients in the simultaneous group had postoperative cardiovascular complications such as myocardial infarction, cardiac arrhythmia, unstable angina, and cardiac failure, whereas 10 patients in the staged group had such complications. After surgery, 17 patients in the simultaneous group and 8 patients in the staged group developed pulmonary infection, pleural effusion, or respiratory failure. A total of 6 patients in the simultaneous group and 4 patients in the staged group had strokes.

After surgery in the simultaneous group, 3 patients developed gastric bleeding, and 1 patient had a cerebral infarction and developed a pulmonary infection. In the staged group, 1 patient had gastric bleeding after surgery. When these major medical complications are combined, the patients in the staged group have a higher rate than the patients in the simultaneous group (23 patients *vs* 41 patients; *P* = 0.031). However, if PE and DVT are added to major medical complications, there is no statistically significant difference between groups (78 patients in the simultaneous group *vs* 33 patients in the staged group; *P* = 0.189). There is also no statistically significant difference between groups for minor medical complications (44 patients in the simultaneous group *vs* 16 patients in the staged group; *P* = 0.707).

### *Surgical Complications*

Superficial infection was the most common surgical complication and occurred more often in the staged group (0.6% *vs* 1.6%, *P* = 0.034). The situation was the same for postoperative dislocation (*P* = 0.049). There was no statistically significant difference between groups for rates of deep infection (0.2% *vs* 0.2%, *P* = 1.000), hematoma (*P* = 0.246), or nerve palsy (*P* = 1.000). When these surgical complications are considered together, patients who underwent staged bilateral THA had higher rates of surgical complications than did patients who underwent simultaneous bilateral THA (*P* = 0.005, Table 2).

### *Readmission, Cumulative Transfusion and Length of Stay*

There was no statistical difference between groups regarding readmission rates (1.6 *vs* 1.8, *P* = 1.000, Table 2). However, patients in the simultaneous group received more cumulative blood transfusions than patients in the staged group (median [interquartile range]: 4 [4–6] *vs* 4 [0–6.5]; *P* < 0.001). However, their LOS was shorter than the LOS for patients in the staged group (median [interquartile range]: 11 [8–14] *vs* 20 [16–24]; *P* < 0.001).

### *Interval Time between Stages of Bilateral Total Hip Arthroplasty*

In the staged bilateral THA group, 57 patients underwent the second THA within 30 days after the first THA, 49 did so at a point between 30 and 90 days afterward, and the remaining 176 did so more than 90 days after the first THA (Table 3). There were no significant differences among these three groups with regard to major medical complications (with or without venous thromboembolism [VTE]), minor medical complications, surgical complications, readmissions, or LOS. However, patients in the staged group who underwent the second THA within 30 days of the first THA required more transfusions than did patients in the other two groups (median [interquartile]: 6 [2–8] *vs* 2 [0–6], *P* = 0.01; 6 [2–8] *vs* 2 [0–6], *P* < 0.001), but there was no significant difference in cumulative transfusions between the patients who underwent the second THA at a point between 30 and 90 days and those who underwent the second procedure after more than 90 days (median [interquartile range]: 2 [0–6] *vs* 2 [0–6], *P* = 0.999).

**Table 3:** Intervals between stages of bilateral total hip arthroplasty

Parameters	Staged group (282 cases)			*P‐*value
	≤30 days (57 cases)	30–90 days (49 cases)	>90 days (176 cases)	
Number of major medical complications without VTE*	4 (7.0)	5 (10.2)	14 (8.0)	0.832
Number of major medical complications including VTE*	7 (12.3)	8 (16.3)	18 (10.2)	0.496
Number of minor medical complications*	3 (5.3)	2 (4.1)	11 (6.3)	0.827
Number of total surgical complications*	4 (7.0)	5 (10.2%)	5 (2.8)	0.098
Number of readmissions*	2 (3.5)	2 (4.1)	1 (0.6)	0.145
Cumulative transfusion (units)†	6 (2–8)	2 (0–6)	2 (0–6)	<0.001
LOS (days)†	21 (15–26.5)	19 (14–23)	19 (14–23)	0.213

* Categorical variables which are presented as frequency with percent in parentheses.

† Continuous variables which are not normally distributed and presented as medians with interquartile ranges in parentheses.

LOS, length of stay; VTE; venous thromboembolism.

## Discussion

The safety of bilateral THA has not been conclusively proven. Previous studies^8^, ^16^, ^17^, ^18^ raised concerns about systemic complications in simultaneous bilateral surgery. Other studies^6^, ^11^ showed that systemic complications of simultaneous surgery were similar to those of unilateral THA. In staged bilateral THA, the cumulative complications of two separate procedures could be higher. However, simultaneous bilateral THA means more blood loss and surgical time for one procedure^8^, ^18^, ^19^, which increases the risks. A meta‐analysis^15^ came out in favor of simultaneous bilateral THA because of reported lower rates of major systemic complications, but the data were highly heterogeneous, with only one study included that listed death as a major systemic complication^7^.

In contrast, our study compared systemic and surgical complications, transfusion rates, readmission rates, and LOS between simultaneous and staged bilateral THA. Although there have been studies with data drawn from large registries^20^, ^21^, ^22^, our study focused on the largest number of cases reported thus far from one center. Analysis of the data for our population showed that with careful selection of patients, simultaneous bilateral THA can result in fewer major medical complications, fewer surgical complications, and shorter hospital stays than for staged bilateral THA, although it can result in a higher transfusion rate.

### *Medical Complications*

Aghayev *et al*.^10^ reported that the incidence of postoperative systemic complications was 7.5% to 10.8% in bilateral THA. However, our study showed a slightly higher rate of 14.1%. We found several reasons why our incidence was higher. First, we performed rigorous checks for VTE postoperatively, and our patients had higher incidences of PE and DVT. Second, our study population consisted of patients from a single hospital database. Third, the indications for hip arthroplasty in our study were different from those in the West (USA), and we had a higher proportion of patients with ankylosing spondylitis and rheumatoid arthritis than other studies did. Choi *et al*.^23^ showed that these patients are at higher risk of developing VTE after total joint arthroplasty. Despite those differences, we believed that our data were representative and were suitable for a comparison of simultaneous versus staged bilateral THA.

Our surgical team, which included an orthopaedic surgeon and an anesthesiologist, chose staged instead of simultaneous bilateral THA mostly in cases in which there was concern about the possibility of major systemic complications, such as cardiovascular failure, pulmonary failure, or stroke. Interestingly, we found no significant difference between our staged group and the simultaneous group in risk for different systems. However, our cumulative results showed that the incidence of major systemic complications (no VTE) was lower in the simultaneous group, similar to the findings in a previous study^10^. Resouli *et al*.^7^ also found that postoperative mortality and complication rates were lower for simultaneous bilateral THA than for staged bilateral THA. The reason for this could have been that the patients undergoing staged THA had more comorbidities. In addition, in our study, patients in the staged THA group were older and had a higher ASA classification than those in the simultaneous THA group. Inneh *et al*.^24^ found that older patients or patients with a higher ASA classification had more major complications. Another reason patients in the staged group had higher complication rates may be that their physical status had not returned to preoperative levels before they underwent the second procedure. Lalmohamed *et al*.^25^ reported that cardiovascular risk can increase even 6 weeks after surgery.

### *Surgical Complications*

We also found that the rate of postoperative surgical complications was higher in the staged group than in the simultaneous group, especially for dislocation and superficial infection. Parvizi *et al*.^11^ established that there was more wound drainage with staged bilateral THA, just as we found. Pulido *et al*.^26^ reported that increased time in surgery was related to wound problems. Duchman *et al*.^27^ reported that when surgery ran longer than 120 minutes, the risk of wound complication increased. They found that with improvements in surgical technique, autologous blood salvage, and the use of tranexamic acid, most patients undergoing simultaneous bilateral THA required less time in surgery and had less blood loss than had been the case before those changes. The higher rate of superficial infection reported for staged THA may be the cumulative result for the two separate procedures.

Previous studies^10^, ^20^ using registry data showed no statistical difference in dislocation rates between simultaneous and staged THA. In our study, however, the staged group had a higher dislocation rate than the simultaneous group did. We had a more conservative rehabilitation protocol for our patients than is used in Western nations (USA). It is possible that patients in our staged group were more active after undergoing the second procedure, which could be why they had more dislocations. Superficial infection and dislocation accounted for most of the surgical complications in the staged group, for which rates were higher than in the simultaneous group. This finding reminds us that we should use a different rehabilitation protocol for patients undergoing staged THA.

### *Readmission, Cumulative Transfusion and Length of Stay*

In our study, cumulative transfusion volume was lower but LOS was longer in the staged group than in the simultaneous group. Some previous studies^16^, ^17^ showed that transfusion volume was lower in staged bilateral THA, as we found. However, another study^11^ showed that patients in staged groups had higher transfusion volumes than did simultaneous groups. Although patients in our simultaneous bilateral group were younger and were more often male than the patients in our staged group, the staged group underwent two separate procedures with an interval between them that could have allowed for better recovery after the first procedure. We believe that this was why transfusion volume was lower in our staged group. As researchers established in previous studies^7^, ^8^, ^13^, ^16^, ^17^, ^18^, ^22^, ^28^, we found that LOS was longer for staged THA, which is not surprising, given that two separate recovery and rehabilitation periods are involved.

### *Interval Time Between Stages of Bilateral Total Hip Arthroplasty*

Garland *et al*.^21^ reported that there was no difference in mortality regardless of the length of interval between stages of bilateral THA. Only one study^10^ has been reported that focused on postoperative systemic complications for different intervals between stages. However, those researchers separated patients into only two groups: ≤6 months and >6 months. Although their data showed that patients who had an interval of >6 months had more complications, they did not provide any reasons because their data was from a registry. Our study showed that patients who underwent the second THA within 30 days had a higher transfusion volume, which could have occurred because those patients had insufficient time after the first procedure to recover from blood loss. However, we found no significant difference between various intervals for medical or surgical complications, readmission rate, or LOS. Thus, it is possible that long intervals between procedures are unnecessary for patients who are good candidates for staged bilateral THA.

Our study had several limitations. First, patients’ selection of simultaneous versus staged bilateral THA was based on the opinion of the medical team and on patients’ wishes. Patients in the simultaneous group were younger, and more members of that group than of the other group were men, which could have been confounding factors. However, this kind of study is difficult to conduct prospectively because of ethics. With the largest cohort reported on so far, our findings may provide some helpful guidance for surgeons in choosing simultaneous versus staged procedures. Second, this was a retrospective cohort study, which means that some information could have been missed. However, the information was prospectively collected and checked carefully. Third, the procedures were performed by several different surgeons, which could have resulted in varying outcomes. However, all of the surgeons were trained in our hospital, and, thus, we believe that their clinical experience levels are similar for primary THA. Finally, because of the different time intervals used for staged bilateral THA, the number of patients in that group was limited. Even though we found no differences regarding outcomes for the various intervals, the small number of patients means that type II errors might have occurred. Additional research is required to confirm our findings.

### *Conclusion*

With careful patient assessment and selection, simultaneous bilateral THA is safe, resulting in fewer surgical and major medical complications than for staged bilateral THA. However, if the interval between procedures is <30 days, the cumulative transfusion rate for simultaneous bilateral THA is higher.

## References

[1] Harris W . The first 50 years of total hip arthroplasty: lessons learned. Clin Orthop Relat Res, 2009, 467: 28–31. 18982399 10.1007/s11999-008-0467-1 PMC2601012

[2] Sayeed SA , Johnson AJ , Jaffe DE , Mont MA . Incidence of contralateral THA after index THA for osteoarthritis. Clin Orthop Relat Res, 2012, 470: 535–540. 21968900 10.1007/s11999-011-2110-9 PMC3254746

[3] Jaffe WL , Charnley J . Bilateral Charnley low‐friction arthroplasty as a single operative procedure: a report of fifty cases. Bull Hosp Joint Dis, 1971, 32: 198–214. 5128234

[4] Kim YH . Bilateral cemented and cementless total hip arthroplasty. J Arthroplasty, 2002, 17: 434–440. 12066272 10.1054/arth.2002.31073

[5] Schäfer M , Elke R , Young JR , Gancs P , Kindler CH . Safety of one‐stage bilateral hip and knee arthroplasties under regional anaesthesia and routine anaesthetic monitoring. J Bone Joint Surg Br, 2005, 87: 1134–1139. 16049253 10.1302/0301-620X.87B8.16207

[6] Stavrakis AI , SooHoo NF , Lieberman JR . Bilateral total hip arthroplasty has similar complication rates to unilateral total hip arthroplasty. J Arthroplasty, 2015, 30: 1211–1214. 25737389 10.1016/j.arth.2015.02.015

[7] Rasouli MR , Maltenfort MG , Ross D , Hozack WJ , Memtsoudis SG , Parvizi J . Perioperative morbidity and mortality following bilateral total hip arthroplasty. J Arthroplasty, 2014, 29: 142–148. 23664280 10.1016/j.arth.2013.04.001

[8] Parvizi J , Tarity TD , Sheikh E , Sharkey PF , Hozack WJ , Rothman RH . Bilateral total hip arthroplasty: one‐stage versus two‐stage procedures. Clin Orthop Relat Res, 2006, 453: 137–141. 17312590 10.1097/01.blo.0000246529.14135.2b

[9] Yoshii T , Jinno T , Morita S , *et al* Postoperative hip motion and functional recovery after simultaneous bilateral total hip arthroplasty for bilateral osteoarthritis. J Orthop Sci, 2009, 14: 161–166. 19337807 10.1007/s00776-008-1303-x

[10] Aghayev E , Beck A , Staub LP , *et al* Simultaneous bilateral hip replacement reveals superior outcome and fewer complications than two‐stage procedures: a prospective study including 1819 patients and 5801 follow‐ups from a total joint replacement registry. BMC Musculoskelet Disord, 2010, 11: 245. 20973941 10.1186/1471-2474-11-245 PMC2987971

[11] Parvizi J , Pour AE , Peak EL , Sharkey PF , Hozack WJ , Rothman RH . One‐stage bilateral total hip arthroplasty compared with unilateral total hip arthroplasty: a prospective study. J Arthroplasty, 2006, 21: 26–31. 16950058 10.1016/j.arth.2006.04.013

[12] Ritter MA , Stringer EA . Bilateral total hip arthroplasty: a single procedure. Clin Orthop Relat Res, 1980, 149: 185–190. 7408301

[13] Berend KR , Lombardi AV Jr , Adams JB . Simultaneous vs staged cementless bilateral total hip arthroplasty: perioperative risk comparison. J Arthroplasty, 2007, 22: 111–115. 10.1016/j.arth.2007.03.043 17823028

[14] Tsiridis E , Pavlou G , Charity J , Tsiridis E , Gie G , West R . The safety and efficacy of bilateral simultaneous total hip replacement: an analysis of 2063 cases. J Bone Joint Surg Br, 2008, 90: 1005–1012. 18669954 10.1302/0301-620X.90B8.20552

[15] Shao H , Chen CL , Maltenfort MG , Restrepo C , Rothman RH , Chen AF . Bilateral total hip arthroplasty: 1‐stage or 2‐stage? A meta‐analysis. J Arthroplasty, 2017, 32: 689–695. 27776901 10.1016/j.arth.2016.09.022

[16] Bhan S , Pankaj A , Malhotra R . One‐ or two‐stage bilateral total hip arthroplasty: a prospective, randomised, controlled study in an Asian population. J Bone Joint Surg Br, 2006, 88: 298–303. 16498000 10.1302/0301-620X.88B3.17048

[17] Alfaro‐Adrián J , Bayona F , Rech JA , Murray DW . One‐ or two‐stage bilateral total hip replacement. J Arthroplasty, 1999, 14: 439–445. 10428224 10.1016/s0883-5403(99)90099-2

[18] Eggli S , Huckell CB , Ganz R . Bilateral total hip arthroplasty: one stage versus two stage procedure. Clin Orthop Relat Res, 1996, 328: 108–118. 8653943

[19] Saito S , Tokuhashi Y , Ishii T , Mori S , Hosaka K , Taniguchi S . One‐ versus two‐stage bilateral total hip arthroplasty. Orthopedics, 2010, 33:8. 20704112 10.3928/01477447-20100625-07

[20] Hooper GJ , Hooper NM , Rothwell AG , Hobbs T . Bilateral total joint arthroplasty the early results from the New Zealand National Joint Registry. J Arthroplasty, 2009, 24: 1174–1177. 19056233 10.1016/j.arth.2008.09.022

[21] Garland A , Rolfson O , Garellick G , Kärrholm J , Hailer NP . Early postoperative mortality after simultaneous or staged bilateral primary total hip arthroplasty: an observational register study from the Swedish hip arthroplasty Register. BMC Musculoskelet Disord, 2015, 16: 77. 25887667 10.1186/s12891-015-0535-0 PMC4393879

[22] Lindberg‐Larsen M , Joergensen CC , Husted H , Kehlet H . Simultaneous and staged bilateral total hip arthroplasty: a Danish nationwide study. Arch Orthop Trauma Surg, 2013, 133: 1601–1605. 23912419 10.1007/s00402-013-1829-z

[23] Choi HK , Rho YH , Zhu Y , Cea‐Soriano L , Aviña‐Zubieta JA , Zhang Y . The risk of pulmonary embolism and deep vein thrombosis in rheumatoid arthritis: a UK population‐based outpatient cohort study. Ann Rheum Dis, 2013, 72: 1182–1187. 22930596 10.1136/annrheumdis-2012-201669

[24] Inneh IA , Lewis CG , Schutzer SF . Focused risk analysis: regression model based on 5314 total hip and knee arthroplasty patients from a single institution. J Arthroplasty, 2014, 29: 2031–2035. 24970581 10.1016/j.arth.2014.05.007

[25] Lalmohamed A , Vestergaard P , Klop C , *et al* Timing of acute myocardial infarction in patients undergoing total hip or knee replacement: a nationwide cohort study. Arch Intern Med, 2012, 172: 1229–1235. 22826107 10.1001/archinternmed.2012.2713

[26] Pulido L , Ghanem E , Joshi A , Purtill JJ , Parvizi J . Periprosthetic joint infection: the incidence, timing, and predisposing factors. Clin Orthop Relat Res, 2008, 466: 1710–1715. 18421542 10.1007/s11999-008-0209-4 PMC2505241

[27] Duchman KR , Pugely AJ , Martin CT , Gao Y , Bedard NA , Callaghan JJ . Operative time affects short‐term complications in total joint arthroplasty. J Arthroplasty, 2017, 32: 1285–1291. 28040399 10.1016/j.arth.2016.12.003

[28] Reuben JD , Meyers SJ , Cox DD , Elliott M , Watson M , Shim SD . Cost comparison between bilateral simultaneous, staged, and unilateral total joint arthroplasty. J Arthroplasty, 1998, 13: 172–179. 9526210 10.1016/s0883-5403(98)90095-x

